# Drug resistance features and S-phase fraction as possible determinants for drug response in a panel of human ovarian cancer xenografts

**DOI:** 10.1054/bjoc.2000.1373

**Published:** 2000-09-04

**Authors:** G M Kolfschoten, T M Hulscher, H M Pinedo, E Boven

**Affiliations:** Department of Medical Oncology, University Hospital Vrije Universiteit, De Boelelaan 1117, Amsterdam, HV 1081, The Netherlands

**Keywords:** ovarian cancer xenografts, drug resistance, *LRP*, S-phase fraction

## Abstract

Multidrug resistance (MDR) and more specifically the expression of P-glycoprotein (Pgp) have been studied extensively in vitro. Unfortunately, it appears that the predictive value of MDR recognized in vitro is mostly an incorrect measure to determine the responsiveness of a particular tumour in the clinic. This misunderstood or overvalued role of MDR might explain the failure of strategies to reverse Pgp function by the use of modulators in solid tumours. To obtain more insight in in vivo drug resistance we investigated a panel of 15 human ovarian cancer xenografts consisting of the most common histological subtypes known in ovarian cancer patients. The response rate to cisplatin, cyclophosphamide and doxorubicin in the xenografts resembled the results of phase II trials with these agents in ovarian cancer patients. This resemblance justifies drug resistance studies in this experimental in vivo human tumour system. We determined the expression levels of *MDR* 1, *MRP* 1, *LRP* and topoisomerase IIα mRNA by the RNase protection assay and the presence of MRP1 and LRP proteins by immunohistochemistry. The S-phase fraction was investigated as a separate parameter by flow cytometry. In none of the 15 ovarian cancer xenografts was *MDR* 1 expression detectable. The expression levels of *MRP* 1 and *LRP* were low to moderate and resembled the presence of the MRP1 and LRP proteins. There was a weak, inverse relationship between the expression levels of *LRP* and sensitivity to cisplatin and cyclophosphamide (*r* = –0.44 and –0.45), but not to doxorubicin. The levels of topoisomerase IIα varied among the xenografts (0.73–2.66) and failed to correlate with doxorubicin resistance (*r* = 0.14). The S-phase fraction, however, showed a relation with the sensitivity to cisplatin (*r* = 0.66). Among the determinants studied in ovarian cancer in vivo, *LRP* mRNA and the S-phase fraction were the best predictive factors for drug response and most specifically for the activity of cisplatin. © 2000 Cancer Research Campaign


British Journal of Cancer (2000) 83(7), 921–927
© 2000 Cancer Research Campaign
doi: 10.1054/ bjoc.2000.1373, available online at http://www.idealibrary.com on IDEAL®

### Drug resistance features and S-phase fraction as possible determinants for drug response in a panel of human ovarian cancer xenografts

**GM Kolfschoten, TM Hulscher, HM Pinedo and E Boven**

Department of Medical Oncology, University Hospital Vrije Universiteit, De Boelelaan 1117, 1081 HV Amsterdam, The Netherlands

**Summary** Multidrug resistance (MDR) and more specifically the expression of P-glycoprotein (Pgp) have been studied extensively in vitro. Unfortunately, it appears that the predictive value of MDR recognized in vitro is mostly an incorrect measure to determine the responsiveness of a particular tumour in the clinic. This misunderstood or overvalued role of MDR might explain the failure of strategies to reverse Pgp function by the use of modulators in solid tumours. To obtain more insight in in vivo drug resistance we investigated a panel of 15 human ovarian cancer xenografts consisting of the most common histological subtypes known in ovarian cancer patients. The response rate to cisplatin, cyclophosphamide and doxorubicin in the xenografts resembled the results of phase II trials with these agents in ovarian cancer patients. This resemblance justifies drug resistance studies in this experimental in vivo human tumour system. We determined the expression levels of *MDR*1, *MRP*1, *LRP* and topoisomerase IIα mRNA by the RNase protection assay and the presence of MRP1 and LRP proteins by immunohistochemistry. The S-phase fraction was investigated as a separate parameter by flow cytometry. In none of the 15 ovarian cancer xenografts was *MDR*1 expression detectable. The expression levels of *MRP*1 and *LRP* were low to moderate and resembled the presence of the MRP1 and LRP proteins. There was a weak, inverse relationship between the expression levels of *LRP* and sensitivity to cisplatin and cyclophosphamide ($r$ = –0.44 and –0.45), but not to doxorubicin. The levels of topoisomerase IIα varied among the xenografts (0.73–2.66) and failed to correlate with doxorubicin resistance ($r$ = 0.14). The S-phase fraction, however, showed a relation with the sensitivity to cisplatin ($r$ = 0.66). Among the determinants studied in ovarian cancer in vivo, *LRP* mRNA and the S-phase fraction were the best predictive factors for drug response and most specifically for the activity of cisplatin. © 2000 Cancer Research Campaign

**Keywords**: ovarian cancer xenografts; drug resistance; *LRP*; S-phase fraction

In advanced ovarian cancer, response to combination chemotherapy may be as high as 70%, but the majority of patients will eventually suffer from recurrent disease. Drug resistance prior to or during the treatment of cancer is a major factor limiting the effectiveness of anticancer agents. The resistance mechanisms in ovarian cancer identified thus far can be divided into three groups: decreased drug accumulation, increased DNA repair, and enhanced detoxification (Johnson et al, 1993). It is difficult to determine the contribution of each of these factors to the outcome of chemotherapy. Human ovarian cancer xenografts might be useful in the elucidation of mechanisms, not only in tumour cells but also at the tissue level, that are associated with drug resistance in vivo.

Glutathione and glutathione-dependent enzymes have been shown to be involved in the detoxification of cisplatin and alkylating agents, which are both well-known drugs for the treatment of ovarian cancer (Schröder et al, 1996). The importance of this detoxification system has been investigated earlier by our group in a panel of 15 human ovarian cancer xenografts (Kolfschoten et al, 2000). A relation between glutathione or the activity of the enzyme glutathione S-transferase and the sensitivity to the anticancer drugs could not be established. This observation confirms clinical findings that suggest that the glutathione detoxification system alone does not play a major role in primary drug resistance in ovarian cancer (Ghazal-Aswad et al, 1996).

Cisplatin conjugated to glutathione can be extruded from the cell by the ATP-dependent glutathione S-conjugate export pump (GS-X) (Ishikawa and Ali-Osman, 1993). The GS-X pump appeared to be functionally related to the multidrug resistance-associated protein (MRP) (Ishikawa et al, 1995). *MRP*1-transfected cells, however, were not cross-resistant against cisplatin (Cole et al, 1994). Moreover, in cisplatin-resistant cells overexpression of *MRP*1 was not evident (Kool et al, 1997). These results indicate that the term GS-X pump might be inappropiate for the description of the function of MRP1. MRP1-overexpressing cells generally show resistance against, for example, anthracyclines, Vinca alkaloids and epipodophyllotoxins (Loe et al, 1996).

For anthracyclines and multiple other hydrophobic natural products it is known that enhanced cellular drug efflux will occur in the presence of the ATP-dependent export pump P170-glycoprotein (Pgp) encoded by the *MDR*1 gene (Roninson, 1992). Both Pgp and MRP1 belong to the ATP-binding cassette (ABC) superfamily of transmembrane transport proteins inducing multidrug resistance. Pharmacological characterization has revealed that the presence of MRP is related to a lower degree of resistance than the presence of Pgp in vitro, i.e. against doxorubicin (Lautier et al, 1996).

More recently, the 110 kD lung resistance protein (LRP) has been discovered which is not a member of the ABC-superfamily.

*Received 10 January 2000*
*Revised 18 May 2000*
*Accepted 22 May 2000*

*Correspondence to:* E Boven

**Table 1** Human ovarian cancer xenografts: histology and volume-doubling time

| Xenograft | Histology | $T^{a}_{d}$ (± SD) |
|---|---|---|
| Ov.Ri(C) | moderately differentiated serous | 10.2 ± 3.5 |
| FKo | moderately differentiated serous | 11.2 ± 3.7 |
| Ov.Gl | poorly differentiated serous | 14.0 ± 5.5 |
| OVCAR-3[b] | poorly differentiated serous | 6.2 ± 2.1 |
| Ov.Sh | poorly differentiated serous | 14.6 ± 4.4 |
| Ov.Pe | moderately differentiated mucinous | 10.5 ± 2.7 |
| Ov.He | moderately differentiated mucinous | 10.4 ± 2.9 |
| Ov.Gr | moderately differentiated mucinous | 14.0 ± 6.7 |
| FMa | poorly differentiated mucinous | 5.9 ± 2.0 |
| FCo | clear cell carcinoma | 7.6 ± 3.2 |
| Ov.Vg | clear cell carcinoma | 8.2 ± 2.9 |
| Ov.Me | carcinosarcoma | 8.1 ± 1.7 |
| MRI-H-207 | undifferentiated | 3.0 ± 0.6 |
| A2780[b] | undifferentiated | 2.9 ± 1.7 |
| H134[b] | undifferentiated | 10.8 ± 2.7 |

[a]$T_d$, mean tumour volume-doubling time in days; [b]established from cell line

LRP has been identified as the major human vault protein (Scheffer et al, 1995). Structural studies of vaults have indicated that these may play a role in cytoplasmic redistribution and the nucleocytoplasmic transport of various substrates. LRP has been reported to be a marker for drug resistance in vitro, both for MDR-related drugs (doxorubicin, vincristine) and for other agents (cisplatin, carboplatin and melphalan) (Izquierdo et al, 1996).

Anthracyclines induce multiple intracellular effects and, thus, resistance in tumour cells can be a multifactorial phenomenon. In addition to the role of Pgp and MRP1 in drug resistance, alterations of the nuclear target enzyme topoisomerase II may be responsible for resistance against doxorubicin (Nielsen et al, 1996). DNA topoisomerase II is a nuclear protein, which is involved in replication of DNA. The protein exists in two isoforms, the 170 kD topoisomerase IIα and the 180 kD IIβ form. Topoisomerase IIα is mainly expressed during the S-phase of the cell cycle and seems to be the preferred target associated with drug resistance.

As none of the investigated resistance features appeared to be a good determinant for tumour response we included a parameter related to cellular kinetics, the S-phase fraction (SPF). In breast cancer patients it has been shown that the number of proliferating cells was a predictive factor for response to neoadjuvant chemotherapy (Chevillard et al, 1996). The S-phase fraction reflects the number of replicating cells. In general, cells with replicating DNA are more vulnerable to the toxic effect of anti-cancer agents.

In our laboratory, we have established a panel of 15 human ovarian cancer xenografts including various histological subtypes as can be recognized in the clinic. We have demonstrated earlier that the xenografts have retained the histological pattern and antigen expression of the tumour of origin (Molthoff et al, 1991). The chemosensitivity of the panel to individual drugs reflects the clinical response rates of similar drugs. Previous collaborative studies showed that human tumour xenografts are useful to predict activity of potential anticancer agents in ovarian cancer (Boven et al, 1992; Langdon et al, 1994). We believe that the panel is relevant for studies on in vivo mechanisms of resistance. In the present experiments we determined the possible contribution of a number of drug resistance features, including the expression of the *MDR*1, *MRP*1 and *LRP* genes, topoisomerase IIα expression and the cell cycle distribution to the chemosensitivity profile of the panel, i.e. to cisplatin, cyclophosphamide and doxorubicin.

### MATERIALS AND METHODS

### Human ovarian cancer xenografts and drug sensitivity

Human ovarian cancer xenografts were grown subcutaneously in the flank of female athymic nude mice (NMRI/Cpb or Hsd: Athymic Nude-*nu*, Harlan, Horst, The Netherlands). The xenografts vary in histological subtype and volume-doubling time and apart from Ov.Sh and Ov.Vg have all been described before (Molthoff et al, 1991) (Table 1). As is typical for human tumour xenografts we have demonstrated that mouse stroma only consists of thin layers whereas in the original ovarian tumours large zones of connective tissue can be detected. In addition, xenograft tissue usually contained less than 10% lactate dehydrogenase of murine origin (Molthoff et al, 1991).

Tumour-bearing mice were treated with cisplatin, cyclophosphamide and doxorubicin at maximum tolerated doses (MTD). Cisplatin (Bristol-Myers Squibb, Woerden, The Netherlands) as a solution of 0.5 mg $ml^{-1}$ was injected i.v. at a dose of 5 mg $kg^{-1}$ every week × 2. Cyclophosphamide (ASTA, Diemen, The Netherlands) dissolved in water 50 mg $ml^{-1}$ was given i.p. twice with a 2-week interval at a dose of 150 mg $kg^{-1}$. Doxorubicin (Pharmacia, Woerden, The Netherlands) was dissolved in water at 2 mg $ml^{-1}$ and was injected i.v. at a dose of 8 mg $kg^{-1}$ every week × 2. At these schedules the mice were required to show a reversible weight-loss of up to 15% as a result of treatment. Drug treatment was started at the time that the tumours had a mean volume of approximately 100 $mm^3$ (designated as day 0). Treatment and control groups consisted of 5–7 mice each. Tumours which failed to reach a volume of 20 $mm^3$ at the start of treatment were considered inevaluable. The increase in tumour volume from the start of treatment ($V_o$) until the value at any given time ($V_t$) was calculated for each tumour and expressed as the relative tumour volume ($V_t$:$V_o$) on the day of measurement. The mean of these values was used to determine the ratio between treated (T) and control (C) tumours (T:C × 100%). The highest percentage of growth inhibition within a period of 5 weeks was considered the optimal growth inhibition. The disappearance of tumours upon treatment for at least 1 month was defined as complete remission. Growth inhibition of the tumours was expressed as 100% – (T:C × 100%). The drug was considered to be active when the growth inhibition was > 50%, very active > 75%, and inactive if inhibition of growth was ≤ 50% (Langdon et al, 1994).

### mRNA gene expression

The expression levels of *MDR*1, *MRP*1, *LRP* and topoisomerase IIα were determined with the RNase protection assay, which was performed as has been described earlier (Giaccone et al, 1995). Measurements were carried out in at least three separate tumours from three different passages per ovarian cancer xenograft. Frozen tumours were pulverized in a microdismembrator and total RNA was isolated with TRIzol (Life Technologies, Breda, The Netherlands). Ten μg of total RNA was hybridized with a specific [$\alpha^{32}$P]CTP-labelled RNA probe complementary to the various mRNAs. Positive controls for *MDR*1 expression were tumour tissue of subline 2780AD generated from the human ovarian cancer cell line A2780 upon continuous exposure to doxorubicin

 © 2000 Cancer Research Campaign

(Hamilton et al, 1984) and the colon cancer cell line COLO320 (Jansen et al, 1995). Tumour tissue of the GLC4/ADR subline, selected with doxorubicin form the human small cell lung cancer cell line GLC4 (Zijlstra et al, 1987), was used as a positive control for *MRP*1 and *LRP* expression.

The [$\alpha^{32}$P]CTP-labelled probe for human *MDR*1 mRNA was obtained by transcription of a 301 bp *MDR*1 cDNA fragment (positions 3500–3801) with SP6 RNA polymerase (Baas et al, 1990). The [$\alpha^{32}$P]CTP-labelled probe of *MRP*1 complementary to the sequences (nucleotides 240–484) at the 5′ end of the *MRP*1 mRNA was transcribed using SP6 RNA polymerase (Zaman et al, 1993). The *LRP* cDNA sequence (nucleotides 2326–2558), inserted in *Apo* I site of pBluescript, was transcribed from *EcoR* I linearized DNA with the use of T7 RNA polymerase (Scheffer et al, 1995). The [$\alpha^{32}$P]CTP-labelled probe specific for topo IIα was transcribed from *Bgl* II-linearized DNA from a Topo IIα-*Xba* I fragment (nucleotides 1277–2440) (Giaccone et al, 1995) in pGEM4 by using SP6 RNA polymerase.

Radiolabelled protected probes were visualized by electrophoresis through a denaturing 6% polyacrylamide gel and followed by autoradiography. In all RNase protection assays a human γ-actin probe was included as a control, for RNA integrity and recovery. The bands were quantitated by densitometric scanning of autoradiographs and the amount of the specific mRNA relative to the amount of γ-actin was calculated.

### Immunohistochemistry

Rat monoclonal antibodies MRPr1 and LMR-5 (Flens et al, 1997) were used to determine the presence of MRP1 and LRP in the ovarian cancer xenografts. Tissue of GLC4/ADR cells grown as a xenograft was included for positive MRPr1 and LMR-5 staining. Cryostat sections from three separate tumours per xenograft were cut (8 mm), air-dried overnight and fixed in ice-cold acetone for 10 min. The slides were blocked with normal rabbit serum for 15 min and incubated with MRPr1 (5 µg ml$^{-1}$) or LMR-5 (5 µg ml$^{-1}$) diluted in PBS/BSA for 60 min at room temperature. The slides were then incubated with a peroxidase-conjugated rabbit anti-rat immunoglobulin (1:25; Dako, Glostrup, Denmark) for 30 min and bound peroxidase was detected with the aminoethyl carbazole substrate kit (Zymed, San Francisco, CA, USA). MRPr1 and LMR-5 staining was expressed as negative (–) to strongly positive (++).

### Cell-cycle distribution

The cell cycle was determined in three separate tumours from three different passages per ovarian cancer xenograft, which were stored at –70°C. Briefly, the frozen tumours were thawed and minced with scissors in cold phosphate-buffered saline (PBS). The cell suspension was filtered through a 160 mm metal mesh, centrifuged at 1200 rpm for 5 min, fixed in a cold 70% ethanol solution, and stored at 4°C until flow cytometric analysis.

Cells were treated with RNase A (Sigma Chemical Co,Zwijndrecht, The Netherlands; 250 mg ml$^{-1}$ in 0.1% Triton X-100) for 20 min at room temperature. Thereafter, staining of cells was performed with propidium iodide (Sigma; final concentration 50 µg ml$^{-1}$) for 20 min at 4°C in the dark. The stained samples, filtered through a 50 µm nylon mesh, were analysed with a fluorescence-activated cell sorter (FACS 420; Becton Dickinson, Mountain View, CA, USA). From each sample, the green fluorescence of $5 \times 10^3$ viable cells was analysed. The S-phase fraction was calculated using LYSIS II software (provided by Becton Dickinson).

### Statistics

Linear regression analysis was used to determine a possible relationship between the sensitivity of the human ovarian cancer xenografts and the drug-resistance features.

### RESULTS

### Chemosensitivity

Tumour-bearing nude mice were treated in the defined schedules with cisplatin, cyclophosphamide and doxorubicin. The sensitivities of the human ovarian cancer xenografts to these drugs are summarized in Table 2. Individual patterns of responsiveness to each of the anticancer agents were found among the different xenografts. Xenografts FKo, Ov.Gr and H134 showed to be resistant against the drugs tested, xenografts Ov.Pe, Ov.He and FCo were poorly responsive and Ov.Sh, Ov.Me, MRI-H-207 and A2780 xenografts were best responsive.

### mRNA gene and protein expression

*MDR*1 mRNA was not detectable in any of the 15 human ovarian cancer xenografts in multiple experiments (Table 3). RNA derived from 2780AD and COLO320 xenografts were included as positive controls, which were indeed strongly positive for *MDR*1 expression (relative expression of 30.0 and 4.0, respectively). As the xenografts of FCo showed a consistent expression of *MRP*1, *LRP* and topoisomerase IIα mRNA, in different passages, these received the value of 1.00 (Table 3). Expression levels in all tumours were calculated as a relative value of the respective FCo xenografts.

The variation of the relative *MRP*1 mRNA levels among the 15 xenografts was small. The expression was 10-fold lower than that in the control small cell lung cancer subline GLC4/ADR (relative

**Table 2** Growth inhibition (%) obtained by single agents in human ovarian cancer xenografts

| Xenograft | Cisplatin[a] | Cyclophosphamide[a] | Doxorubicin[a] |
|---|---|---|---|
| Ov.Ri(C) | 77 (++) | 57 (+) | 68 (+) |
| FKo | 42 (–) | 4 (–) | 8 (–) |
| Ov.Gl | 77 (++) | 57 (+) | 59 (+) |
| OVCAR-3 | 85 (++) | 60 (+) | 44 (–) |
| Ov.Sh | 94 (++) | 94 (++) | 91 (++) |
| Ov.Pe | 37 (–) | 62 (+) | 54 (+) |
| Ov.He | 30 (–) | 10 (–) | 66 (+) |
| Ov.Gr | 13 (–) | 10 (–) | 0 (–) |
| FMa | 75 (+) | 64 (+) | 53 (+) |
| FCo | 45 (–) | 39 (–) | 67 (+) |
| Ov.Vg | 57 (+) | 66 (+) | 37 (–) |
| Ov.Me | 80 (++) | 97 (++) | 69 (+) |
| MRI-H-207 | CR[b] (++) | CR (++) | CR (++) |
| A2780 | 54 (+) | 88 (++) | 81 (++) |
| H134 | 23 (–) | 10 (–) | 41 (–) |

[a]growth inhibition < 50% –; 50%–75% +; ≥ 75% ++; [b]CR = complete remission

© 2000 Cancer Research Campaign 

**Table 3** Drug resistance features of 15 human ovarian cancer xenografts

| Xenografts | *MDR*1 mRNA | *MRP*1 mRNA (±SEM) | MRPr1 staining | *LRP* mRNA (±SEM) | LMR-5 staining | Topo IIa mRNA (±SEM) | S-phase fraction % (±SEM) |
|---|---|---|---|---|---|---|---|
| Ov.Ri(C) | n.d.[a] | 1.53 ± 0.32 | ±/+ | 1.29 ± 0.33 | –/± | 2.66 ± 0.20 | 20.5 ± 3.0 |
| FKo | n.d. | 1.49 ± 0.22 | – | 1.18 ± 0.18 | –/± | 1.52 ± 0.11 | 24.8 ± 5.2 |
| Ov.Gl | n.d. | 1.11 ± 0.15 | – | 1.27 ± 0.23 | –/± | 1.29 ± 0.30 | 22.7 ± 2.5 |
| OVCAR-3 | n.d. | 0.60 ± 0.13 | + | 0.80 | – | 1.74 ± 0.16 | 31.2 ± 1.4 |
| Ov.Sh | n.d. | 0.69 ± 0.18 | ± | 1.26 ± 0.12 | +[b] | 1.33 ± 0.08 | 24.9 ± 2.0 |
| Ov.Pe | n.d. | 0.81 ± 0.11 | + | 2.20 ± 0.20 | ++ | 1.12 ± 0.06 | 2.4 ± 0.8 |
| Ov.He | n.d. | 0.70 ± 0.16 | – | 2.14 ± 0.27 | + | 0.73 ± 0.10 | 16.6 ± 3.9 |
| Ov.Gr | n.d. | 1.08 ± 0.20 | – | 1.00 ± 0.12 | ±/+[b] | 1.18 ± 0.18 | 10.7 ± 2.1 |
| FMa | n.d. | 0.99 ± 0.11 | – | 0.94 ± 0.10 | ±/+ | 2.06 ± 0.23 | 17.5 ± 1.0 |
| FCo | n.d. | 1.0 | + | 1.0 | +[b] | 1.0 | 8.8 ± 1.6 |
| Ov.Vg | n.d. | 1.61 ± 0.31 | +/++ | 1.24 ± 0.30 | – | 1.09 ± 0.10 | 13.3 ± 2.2 |
| Ov.Me | n.d. | 1.02 ± 0.24 | –/± | 0.79 ± 0.11 | – | 1.28 ± 0.15 | 12.6 ± 0.7 |
| MRI-H-207 | n.d. | 1.22 ± 0.17 | – | 0.68 ± 0.18 | – | 1.64 ± 0.14 | 39.4 ± 4.7 |
| A2780 | n.d. | 1.13 ± 0.18 | – | 0.37 ± 0.07 | – | 1.91 ± 0.25 | 23.4 ± 2.7 |
| H134 | n.d. | 0.97 ± 0.16 | – | 1.49 ± 0.34 | – | 1.70 ± 0.23 | 27.0 ± 2.2 |

[a] n.d. = not detectable; [b]heterogeneous staining

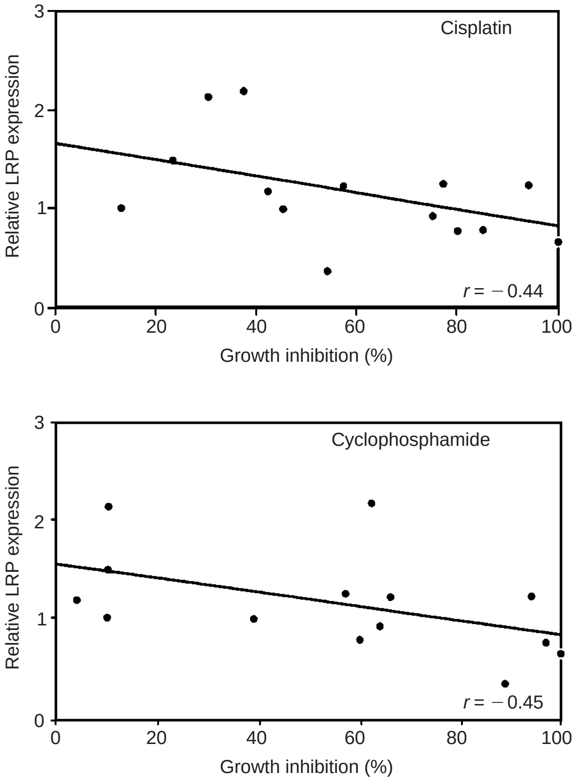

**Figure 1** Relation between *LRP* expression and the growth inhibition (%) induced by cisplatin or cyclophosphamide in 15 human ovarian cancer xenografts. Mice bearing well-established tumours were injected with cisplatin i.v. at a dose of 5 mg $kg^{-1}$ every week × 2 or were treated with cyclophosphamide twice i.p. with a 2-week interval at a dose of 150 mg $kg^{-1}$. The *r* value was calculated by linear regression analysis.

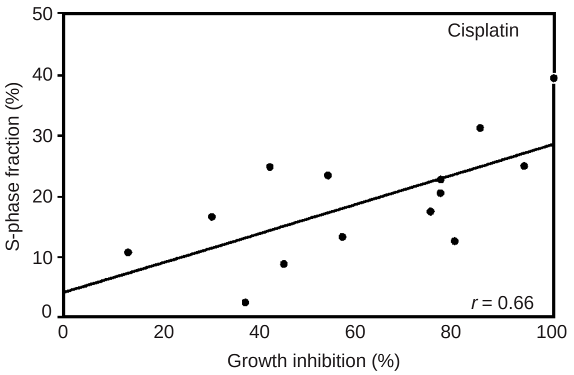

**Figure 2** Relation between the S-phase fraction and growth inhibition (%) induced by cisplatin in human 15 human ovarian cancer xenografts. Mice bearing well-established tumours were injected with cisplatin i.v. at a dose of 5 mg $kg^{-1}$ every week × 2. The *r* value was calculated by linear regression analysis.

expression of 10.7; GLC4 relative expression 0.97). The expression levels of the MRP1 protein were low to moderate in 50% of the xenografts as determined by immunohistochemistry. The control GLC4/ADR xenograft showed strongly positive staining. Xenografts with high levels of *MRP*1 mRNA expression also showed detectable levels of the MRP1 protein, except for FKo tumours. Apart from FKo, in other drug-resistant xenografts Ov.Gr and H134, MRP1 staining was also negative. In general, no relation was present between the expression levels of *MRP*1 mRNA and the anti-tumour effects of cisplatin or doxorubicin ($r$ = 0.01 and 0.24, respectively).

Low to moderate levels of *LRP* mRNA expression were found among the 15 xenografts. *LRP* mRNA levels varied between 0.37 in A2780 xenografts and 2.20 in the Ov.Pe xenograft. Expression of *LRP* mRNA in the control GLC4/ADR xenografts was moderate (1.39), while this was low in GLC4 (0.73). In 50% of the tumour samples the LRP protein could be detected (Table 3). The control GLC4/ADR tissue also showed low staining. LRP protein expression correlated with the expression levels of *LRP* mRNA. The drug-resistant xenografts FKo and Ov.Gr and the poorly responsive xenografts Ov.Pe, Ov.He and FCo showed moderate to high amounts of LRP protein and moderate levels of *LRP* mRNA expression. When the expression levels of *LRP* were compared with cisplatin and cyclophosphamide sensitivity, there appeared to be a weak, inverse relation ($r$ = – 0.44 and –0.45, respectively) (Figure 1), but this was not the case for doxorubicin ($r$ = – 0.15).

*© 2000 Cancer Research Campaign*

Topoisomerase IIα mRNA was detectable in all xenografts. Expression levels varied between 0.73 in Ov.He xenografts and 2.66 in Ov.Ri(C) xenografts. The resistant xenografts FKo and Ov.Gr expressed relatively low levels of topoisomerase IIα mRNA. H134 tumours, however, contained moderate levels of topoisomerase IIα mRNA. There was no relation between topoisomerase IIα expression and sensitivity to doxorubicin ($r$ = 0.14).

### Cell-cycle distribution

$G_1$, S and $G_2$/M phases of the xenografts were identified by flow cytometric determination of cellular DNA content after propidium iodide staining. Particular attention was paid to the S-phase fraction, as in breast cancer patients a high fraction of cells in the S- or S+$G_2$-phase has been shown to predict for drug response (Remvikos et al, 1989; Spyratos et al, 1992; Chevillard et al, 1996). The S-phase fraction varied between 2.4% and 39.4% in the different xenografts (Table 3). A relatively low S-phase fraction was found in the resistant xenograft Ov.Gr xenograft. FKo and H134 tumours contained a high number of cells in S-phase. Overall, there was a clear relation between the S-phase fraction and the sensitivity to cisplatin ($r$ = 0.66) (Figure 2), while this was not the case for cyclophosphamide and doxorubicin ($r$ = 0.31 and 0.34, respectively).

### DISCUSSION

Drug resistance in general remains a barrier to the successful treatment of solid tumours. Most research has been dedicated towards the role of multi-drug-resistance (MDR) expressed by Pgp, including measures to overcome MDR. Tumour heterogeneity in the expression of MDR features may be a reason for the disappointing results obtained in the treatment of solid tumours with modulators of Pgp (Pinedo and Giaccone, 1995). The negative data can also be due to the drug interaction between MDR modulators and anti-cancer agents resulting in increased toxicity. Another misleading factor is the misunderstood or overvalued role ascribed to MDR from results obtained in vitro. A limitation of in vitro drug resistance studies is the use of monolayer cultures of tumour cells, which are known to be more vulnerable to drugs than the same cells grown as solid tumours in vivo. This can be explained by factors such as insufficient drug penetration, a reduced growth fraction, or a decreased sensitivity mediated by cell–cell interactions. In addition, drug resistance is very likely a multifactorial phenomenon. Therefore, we determined a number of resistance features in a large panel of human ovarian cancer xenografts that was characterized earlier for drug sensitivity.

Several groups have shown that human tumour xenografts are useful for drug screening and predictive for clinical drug response (Boven et al, 1992; Langdon et al, 1994). In the panel of 15 human ovarian cancer xenografts we have demonstrated that the efficacy of cisplatin, cyclophosphamide and doxorubicin predicted for the response rates of these drugs described in ovarian cancer patients (Neijt et al, 1984). Briefly, by applying a cut-off growth inhibition of > 75% it was shown that 40% of the xenografts responded to cisplatin, which was close to the 48% reported in patients. For cyclophosphamide the respective values were 27% and 40% and for doxorubicin 20% and 35%. In this respect, we consider our panel of human ovarian cancer xenografts of value to detect a possible relation between the efficacy of anti-cancer agents and drug resistance features. Moreover, host stroma contents are limited in xenograft tissue which offers a better basis for tumour tissue analysis than human tumour tissue invaded by thick layers of stromal cells.

In none of the ovarian cancer xenografts could *MDR*1 expression be measured using the RNase protection assay, while expression of *MDR*1 was clearly detectable in the positive controls. In general, the frequency and intensity of *MDR*1 expression or the presence of Pgp in samples of ovarian cancer is low. In untreated patient's samples the expression rates for Pgp, determined by immunohistochemistry, were 17%, 16% and 7% (Rubin et al, 1990; Izquierdo et al, 1995; Arts et al, 1999). In Northern blot analysis *MDR*1 expression was undetectable in untreated patient's samples, while only 6% of samples of treated patients were positive (Bourhis et al, 1989). The percentage of positive *MDR*1 expression increased to 81% (Noonan et al, 1990) and 36% (Kavallaris et al, 1996) with the more sensitive polymerase chain reaction (PCR). In our xenografts the expression levels of *MDR*1 were too low to detect with the RNase protection assay, which is in agreement with Bourhis et al (1989). Whether low quantities of *MDR*1, detectable only with PCR, are clinically meaningful is questionable.

No relation could be established between *MDR*1 mRNA expression levels or the amount of MRP1 protein and the sensitivity to doxorubicin or cisplatin. For cisplatin, this finding is consistent with the inability of the *MRP*1 gene to confer resistance against this drug, as has been demonstrated in transfection experiments (Cole et al, 1994). In ovarian cancer patients *MRP*1 expression failed to affect response to chemotherapy or disease-free survival (lzquierdo et al, 1995; Kavallaris et al, 1996; Arts et al, 1999). It has been shown that the GS-X pump is able to transport cisplatin conjugated to glutathione out of the cell (Ishikawa and Ali-Osman, 1993). Although MRP1 appeared to be functionally related to the GS-X pump, there are conflicting results on whether MRP1 plays a role in platinum resistance. The precise role of MRP1 in clinical drug resistance and, more specifically, in transporting glutathione-drug conjugates out of the cell has yet to be delineated.

The drug-resistant and partly responsive human ovarian cancer xenografts contained moderate to high levels of *LRP* mRNA and LRP protein, and a weak, inverse relationship with the sensitivity to cisplatin and cyclophosphamide ($r$ = –0.44 and –0.45, respectively), but not in the case of doxorubicin. In 61 unselected cancer cell lines, it has been described that LRP expression was associated with relative resistance not only against carboplatin and cisplatin, but also against melphalan and doxorubicin (Izquierdo et al, 1996). This finding for both MDR and non-MDR drugs supports the coexistence of different resistance mechanisms. In 57 patients with advanced ovarian cancer stage III/IV analysed by Izquierdo et al (1995), LRP expression levels appeared to be an independent prognostic factor of poor response to chemotherapy and for shorter progression-free and overall survival. Of interest, LRP expression was also associated with an inferior response to induction chemotherapy in acute myeloid leukaemia (List et al, 1996), as well as to conventional dose melphalan in multiple myeloma (Raaijmakers et al, 1998). In contrast, in a recent study in 115 patients with ovarian cancer stages I–IV, LRP expression was more frequently observed in patients with early stage, lower grade, and smaller residual tumour lesions (Arts et al, 1999), known to be favourable prognostic factors in this type of disease. In that study, LRP did not appear to be an independent prognostic factor for

© 2000 Cancer Research Campaign 

chemotherapy response or progression-free survival in 59 evaluable patients. Arts et al (1999) have speculated that the apparent difference between patient populations in their study and that of Izquierdo et al (1995) are responsible for the conflicting results.

Topoisomerase IIα mRNA levels varied 3.5-fold among the human ovarian cancer xenografts and were not related to the sensitivity to doxorubicin. Inter-tumour variability of the topoisomerase IIα mRNA level and activity of the enzyme has been described in ovarian cancer samples of patients (Van der Zee et al, 1991; Cornarotti et al, 1996). Both groups have indicated a reduction in topoisomerase IIα expression/activity after cisplatin-based therapy, but the clinical utility of this determinant as an indicator of drug resistance has been questioned. In addition, Hamaguchi et al (1993) have shown that cross-resistance against doxorubicin in cisplatin-resistant ovarian cancer cell lines was not due to alterations in topoisomerase II mRNA levels, but merely the result of an increased amount of glutathione.

Most studies on the predictive value of the cell-cycle distribution for the efficacy of chemotherapy have been carried out in patients with breast cancer. A significant correlation has been observed between the S-phase fraction and the response to multiple anticancer agents given as neoadjuvant treatment before surgery of the breast (Remvikos et al, 1989; Spyratos et al, 1992; Chevillard et al, 1996). One study in soft tissue sarcoma patients supported this finding, where seven out of 10 patients with S-phase fractions > 6% showed a response to preoperative doxorubicin and cisplatin. In contrast, only three of 12 soft tissue sarcoma patients with a lower S-phase fraction responded to treatment (Schmidt et al, 1993). In agreement with these data, the S-phase fraction in our human ovarian cancer xenografts showed a relation with the sensitivity to cisplatin ($r$ = 0.66).

Multiple drug-resistance features may account for drug resistance in vivo, while single mechanisms have mostly been described in selected cell lines in vitro. The contribution of each individual drug-resistance feature to the response observed in a patient is still unclear. Clinically, cellular determinants of influence on the response rate are also difficult to define due to treatment with multiple agents. Our panel of 15 human ovarian cancer xenografts resembles both clinical histological subtypes and chemosensitivity of ovarian cancer. In agreement with the results obtained in patient's samples, we could barely establish a relationship between individual drug-resistance features and drug response, in the case of single agents in our panel of 15 human ovarian cancer xenografts. Other cellular features may be involved in the complex phenotype of drug resistance in general, such as DNA repair mechanisms contributing to resistance against alkylating agents, or variations in the apoptotic pathway causing a poor drug response. Moreover, we recently observed that the degree of tumour vascularization is of influence on the growth inhibition induced by cisplatin (Duyndam et al, 2000). In vitro findings should be tested in an appropriate human tumour system in vivo, such as a panel of human tumour xenografts, to determine the prognostic relevance of drug resistance features for cancer treatment in the clinic.

### REFERENCES

Arts HJG, Katsaros D, De Vries EGE, Massobrio M, Genta F, Danese S, Arisio R, Scheper RJ, Kool M, Scheffer GL, Willemse PHB, Van der Zee AGJ and Suurmeijer AJH (1999) Drug resistance-associated markers P-glycoprotein, multidrug resistance- associated protein 1, multidrug resistance-associated protein 2, and lung resistance protein as prognostic factors in ovarian carcinoma. *Clin Cancer Res* **5**: 2798–2805

Baas F, Jongsma APM, Broxterman HJ, Arceci RJ, Housman D, Scheffer GL, Riethorst A, Van Groenigen M, Nieuwint AWM and Joenje H (1990) Non-P-glycoprotein mediated mechanism for multidrug resistance precedes P-glycoprotein expression during in vitro selection for doxorubicin resistance in a human lung cancer cell line. *Cancer Res* **50**: 5392–5398

Bourhis J, Goldstein LJ, Riou G, Pastan I, Gottesman MM and Benard J (1989) Expression of a human multidrug resistance gene in ovarian carcinomas. *Cancer Res* **49**: 5062–5065

Boven E, Winograd B, Berger DP, Dumont MP, Braakhuis BJM, Fodstad O, Langdon SP and Fiebig HH (1992) Phase II preclinical drug screening in human tumour xenografts: a first European multicenter collaborative study. *Cancer Res* **52**: 5940–5947

Chevillard S, Pouillart P, Beldjord C, Asselain B, Beuzeboc P, Magdelenat H and Vielh P (1996) Sequential assessment of multidrug resistance phenotype and measurement of S-phase fraction as predictive markers of breast cancer response to neoadjuvant chemotherapy. *Cancer* **77**: 292–300

Cole SPC, Sparks KE, Fraser K, Loe DW, Grant CE, Wilson GM and Deeley RG (1994) Pharmacological characterization of multidrug resistant MRP-transfected human tumour cells. *Cancer Res* **54**: 5902–5910

Cornarotti M, Capranico G, Bohm S, Oriana S, Spatti GB, Mariani L, Ballabio G and Zunino F (1996) Gene expression of DNA topoisomerases I, II alpha and II beta and response to cisplatin-based chemotherapy in advanced ovarian carcinoma. *Int J Cancer* **67**: 479–484

Duyndam MCA, Hilhorst MCGW, Verheul HMW, Pinedo HM and Boven E (2000) $VEGF_{165}$ expression differentially affects the growth inhibition induced by anticancer agents in human ovarian cancer xenografts. *Keystone Symposium on angiogenesis*. Salt Lake City, March 2000, abst

Flens MJ, Scheffer GL, Van der Valk P, Broxterman HJ, Eijdems EWHM, Huysmans ACLM, Izquierdo MA and Scheper RJ (1997) Identification of novel drug resistance-associated proteins by a panel of rat monoclonal antibodies. *Int J Cancer* **73**: 249–257

Ghazal-Aswad S, Hogarth L, Hall AG, George M, Sinha DP, Lind M, Calvert AH, Sunter JP and Newell DR (1996) The relationship between tumour glutathione concentration, glutathione S-transferase isoenzyme expression and response to single agent carboplatin in epithelial ovarian cancer patients. *Br J Cancer* **74**: 468–473

Giaccone G, Van Ark-Otte J, Scagliotti G, Capranico G, Van der Valk P, Rubio G, Dalesio O, Lopez R, Zunino F, Walboomers J and Pinedo HM (1995) Differential expression of DNA topoisomerases in non-small cell lung cancer and normal lung. *Biochim Biophys Acta* **1264**: 337–346

Hamaguchi K, Godwin AK, Yakushiji M, O-Dwyer PJ, Ozols RF and Hamilton TC (1993) Cross-resistance to diverse drugs is associated with primary cisplatin resistance in ovarian cancer cell lines. *Cancer Research* **53**: 5225–5232

Hamilton TC, Young RC and Ozols RF (1984) Experimental model systems of ovarian cancer: applications to the design and evaluation of new treatment approaches. *Semin Oncol* **11**: 285–298

Ishikawa T and Ali-Osman F (1993) Glutathione-associated cis-diamminedichloroplatinum(II) metabolism and ATP-dependent efflux from leukemia cells. Molecular characterization of glutathione-platinum complex and its biological significance. *J Biol Chem* **268**: 20116–20125

Ishikawa T, Akimaru K, Kuo MT, Priebe W and Suzuki M (1995) How does the MRP/GS-X pump export doxorubicin? [letter]. *J Natl Cancer Inst* **87**: 1639–1640

Izquierdo MA, Van der Zee AGJ, Vermorken JB, Van der Valk P, Belien JAM, Giaccone G, Scheffer GL, Flens MJ, Pinedo HM, Kenemans P, Meijer CJLM, De Vries EGE and Scheper RJ (1995) Drug resistance-associated marker Lrp for prediction of response to chemotherapy and prognoses in advanced ovarian carcinoma. *J Natl Cancer Inst* **87**: 1230–1232

Izquierdo MA, Shoemaker RH, Flens MJ, Scheffer GL, Wu L, Prather TR and Scheper RJ (1996) Overlapping phenotypes of multidrug resistance among panels of human cancer-cell lines. *Int J Cancer* **65**: 230–237

Jansen WJM, Pinedo HM, Van der Wilt CL, Feller N, Bamberger U and Boven E (1995) The influence of BIBW22BS, a dipyridamole derivative, on the antiproliferative effects of 5-fluorouracil, methotrexate and gemcitabine in vitro and in human tumour xenografts. *Eur J Cancer* **31A**: 2313–2319

Johnson SW, Ozols RF and Hamilton TC (1993) Mechanisms of drug resistance in ovarian cancer. *Cancer* **71**: 644–649

Kavallaris M, Leary JA, Barrett JA and Friedlander ML (1996) MDR1 and multidrug resistance-associated protein (MRP) gene expression in epithelial ovarian tumours. *Cancer Lett* **102**: 7–16

Kolfschoten GM, Pinedo HM, Scheffer PG, Schlüper HMM, Erkelens CAM and Boven E (2000) Development of a panel of 15 human ovarian cancer

*© 2000 Cancer Research Campaign*

xenografts for drug screening and determination of the role of the glutathione detoxification system. *Gynecol Oncol* **76**: 362–368

Kool M, De Haas M, Scheffer GL, Scheper RJ, Van Eijk MJT, Juijn JA, Baas F and Borst P (1997) Analysis of expression of cMOAT (MRP2), MRP3, MRP4, and MRP5, homologues of the multidrug resistance-associated protein gene (MRP1), in human cancer cell lines. *Cancer Res* **57**: 3537–3547

Langdon SP, Hendriks HR, Braakhuis BJM, Pratesi G, Berger DP, Fodstad O, Fiebig HH and Boven E (1994) Preclinical phase II studies in human tumour xenografts: a European multicenter follow-up study. *Ann Oncol* **5**: 415–422

Lautier D, Canitrot Y, Deeley RG and Cole SP (1996) Multidrug resistance mediated by the multidrug resistance protein (MRP) gene. *Biochem Pharmacol* **52**: 967–977

List AF, Spier CS, Grogan TM, Johnson C, Roe DJ, Greer JP, Wolff SN, Broxterman HJ, Scheffer GL, Scheper RJ and Dalton WS (1996) Overexpression of the major vault transporter protein lung-resistance protein predicts treatment outcome in acute myeloid leukemia. *Blood* **87**: 2464–2469

Loe DW, Deeley RG and Cole SPC (1996) Biology of the multidrug resistance-associated protein, MRP. *Eur J Cancer* **32A**: 945–957

Molthoff CFM, Calame JJ, Pinedo HM and Boven E (1991) Human ovarian cancer xenografts in nude mice: characterization and analysis of antigen expression. *Int J Cancer* **47**: 72–79

Neijt JP, Ten Bokkel Huinink WW, Van der Burg MEL, Van Oosterom AT, Vriesendorp R and Pinedo HM (1984) Current status of systemic chemotherapy in the treatment of advanced ovarian cancer with emphasis on CHAP-5. *Radiother Oncol* **2**: 19–29

Nielsen D, Maare C and Skovsgaard T (1996) Cellular resistance to anthracyclines. *Gen Pharmacol* **27**: 251–255

Noonan KE, Beck C, Holzmayer TA, Chin JE, Wunder JS, Andrulis IL, Gazdar AF, Willman CL, Griffith B, Von Hoff DD and Roninson IB (1990) Quantitative analysis of MDR1 (multidrug resistance) gene expression in human tumours by polymerase chain reaction. *Proc Natl Acad Sci USA* **87**: 7160–7164

Pinedo HM and Giaccone G (1995) P-glycoprotein – a marker of cancer-cell behavior [editorial; comment]. *N Engl J Med* **333**: 1417–1419

Raaijmakers HG, Izquierdo MA, Lokhorst HM, De Leeuw C, Belien JA, Bloem AC, Dekker AW, Scheper RJ and Sonneveld P (1998) Lung-resistance-related protein expression is a negative predictive factor for response to conventional low but not to intensified dose alkylating chemotherapy in multiple myeloma. *Blood* **91**: 1029–1036

Remvikos Y, Beuzeboc P, Zajdela A, Voillemot N, Magdelenat H and Pouillart P (1989) Correlation of pretreatment proliferative activity of breast cancer with the response to cytotoxic chemotherapy. *J Natl Cancer Inst* **81**: 1383–1387

Roninson IB (1992) The role of the MDR1 (P-glycoprotein) gene in multidrug resistance in vitro and in vivo. *Biochem Pharmacol* **43**: 95–102

Rubin SC, Finstad CL, Hoskins WJ, Saigo PE, Provencher DM, Federici MG, Hakes TB, Markman M, Reichman BS, Lloyd KO and Lewis JL (1990) Expression of P-glycoprotein in epithelial ovarian cancer: evaluation as a marker of multidrug resistance. *Am J Obstet Gynecol* **163**: 69–73

Scheffer GL, Wijngaard PLJ, Flens MJ, Izquierdo MA, Slovak ML, Pinedo HM, Meijer CJLM, Clevers HC and Scheper RJ (1995) The drug resistance-related protein LRP is the human major vault protein. *Nat Med* **1**: 578–582

Schmidt RA, Conrad EU, Collins C, Rabinovitch P and Finney A (1993) Measurement and prediction of the short-term response of soft tissue sarcomas to chemotherapy. *Cancer* **72**: 2593–2601

Schröder CP, Godwin AK, O'Dwyer PJ, Tew KD, Hamilton TC and Ozols RF (1996) Glutathione and drug resistance. *Cancer Invest* **14**: 158–168

Spyratos F, Briffod M, Tubiana-Hulin M, Andrieu C, Mayras C, Pallud C, Lasry S and Rouesse J (1992) Sequential cytopunctures during preoperative chemotherapy for primary breast carcinoma. II. DNA flow cytometry changes during chemotherapy, tumor regression, and short-term follow-up. *Cancer* **69**: 470–475

Van der Zee AGJ, Hollema H, De Jong S, Boonstra H, Gouw A, Willemse PHB, Zijlstra JG and De Vries EGE (1991) P-glycoprotein expression and DNA topoisomerase I and II activity in benign tumors of the ovary and in malignant tumors of the ovary, before and after platinum/cyclophosphamide chemotherapy. *Cancer Res* **51**: 5915–5920

Zaman GJR, Versantvoort CHM, Smit JJM, Eijdems EWHM, De Haas M, Smith AJ, Broxterman HJ, Mulder NH, De Vries EGE, Baas F and Borst P (1993) Analysis of the expression of MRP, the gene for a new putative transmembrane drug transporter, in human multidrug resistant lung cancer cell lines. *Cancer Res* **53**: 1747–1750

Zijlstra JG, De Vries EGE and Mulder NH (1987) Multifactorial drug resistance in an adriamycin-resistant human small cell lung carcinoma cell line. *Cancer Res* **47**: 1780–1784

*© 2000 Cancer Research Campaign*
